# Salt Tolerance and Na Allocation in *Sorghum bicolor* under Variable Soil and Water Salinity

**DOI:** 10.3390/plants9050561

**Published:** 2020-04-28

**Authors:** Roberta Calone, Rabab Sanoubar, Carla Lambertini, Maria Speranza, Livia Vittori Antisari, Gilmo Vianello, Lorenzo Barbanti

**Affiliations:** Department of Agricultural and Food Sciences (DISTAL), University of Bologna, 40127 Bologna, Italy; roberta.calone3@unibo.it (R.C.); rabab.sanoubar@unibo.it (R.S.); maria.speranza@unibo.it (M.S.); livia.vittori@unibo.it (L.V.A.); gilmo.vianello@unibo.it (G.V.); lorenzo.barbanti@unibo.it (L.B.)

**Keywords:** *Sorghum bicolor*, salinity, salt leaching, sodium translocation, element balance

## Abstract

Salinity is a major constraint for plant growth in world areas exposed to salinization. *Sorghum bicolor* (L.) Moench is a species that has received attention for biomass production in saline areas thanks to drought and salinity tolerance. To improve the knowledge in the mechanisms of salt tolerance and sodium allocation to plant organs, a pot experiment was set up. The experimental design combined three levels of soil salinity (0, 3, and 6 dS m^−1^) with three levels of water salinity (0, 2–4, and 4–8 dS m^−1^) and two water regimes: no salt leaching (No SL) and salt leaching (SL). This latter regime was carried out with the same three water salinity levels and resulted in average +81% water supply. High soil salinity associated with high water salinity (HSS-HWS) affected plant growth and final dry weight (DW) to a greater extent in No SL (−87% DW) than SL (−42% DW). Additionally, HSS-HWS determined a stronger decrease in leaf water potential and relative water content under No SL than SL. HSS-HWS with No SL resulted in a higher Na bioaccumulation from soil to plant and in translocation from roots to stem and, finally, leaves, which are the most sensitive organ. Higher water availability (SL), although determining higher salt input when associated with HWS, limited Na bioaccumulation, prevented Na translocation to leaves, and enhanced selective absorption of Ca vs. Na. At plant level, higher Na accumulation was associated with lower Ca and Mg accumulation, especially in No SL. This indicates altered ion homeostasis and cation unbalance.

## 1. Introduction

Salinity is a major cause of soil degradation in agricultural land worldwide, and arid and semi-arid climate zones are the most affected [1]. In Europe, saline soils account for about 3.8 million ha, mostly located in the Mediterranean area [2]. Coastal areas are experiencing groundwater salinization due to seawater intrusion into the shallow water table [3,4]. This condition is progressively leading to secondary salinization [5], with consequent loss of soil fertility and crop productivity [6,7]. 

In particular, salinity affects soil biodiversity, microbial activities, and biochemical cycles, interfering with soil respiration, organic residue decomposition, nitrification, and denitrification [8]. Additionally, salinity alters the soil physicochemical properties, leading to organic matter reduction and sodification, with consequent clay particle dispersion and loss in aggregate stability. This makes soil less structured and undermines soil hydraulic conductivity and water storage/drainage capacity, increasing surface runoff and wind erosion vulnerability [9]. 

Under salt stress conditions, stronger, i.e., more negative, osmotic potential in the soil solution affects seed germination, seedling establishment, and crop growth [10]. According to the biphasic model proposed by Munns [11], plant response to salinity is articulated in two phases: the first phase is an immediate growth reduction due to osmotic effect, which is similar to what happens under water stress; the second phase is a slower effect due to progressive salt ion accumulation. The first osmotic effect is due to the decrease in the leaf osmotic potential, which is necessary to counterbalance the decrease in the soil osmotic potential and allow plants to take up water and nutrients. The subsequent ion-specific effect, instead, is the consequence of toxic build-up of saline ions in plant organs, causing nutritional disorders, membrane disorganization, and oxidative stress, followed by reduction in cell division and expansion. Generally, salt-tolerant plants differ from the sensitive ones, especially in their ability to control salt accumulation and endure its deleterious effect [12,13].

Under climatic change, soil salinization is expected to increase in the Mediterranean region, because extreme heat and drought events are becoming more and more frequent [14,15].

Many strategies are envisaged to preserve soil productivity, based on irrigation management and choice of salt-tolerant species [16,17,18]. In particular, salt leaching through excess irrigation is a practice often used to leach soluble salts out of the root zone. However, when only brackish water is available, the efficiency of this method and its effect on plant growth are debated.

A good candidate for investigations on salinity is *Sorghum bicolor* (L.) Moench (Poaceae), which is commonly referred to as sorghum. Thanks to C_4_ metabolism, sorghum can sustain photosynthetic activity and dry matter production in stressful conditions such as high temperature, drought and salinity [19,20]. Owing to this, sorghum is the fifth most cultivated cereal crop in arid and semiarid world regions [21] and is regarded as a tolerant crop plant for marginal conditions, including saline soils.

Sorghum is believed to tolerate soil and water salinity up to 6.8 and 4.5 dS m^−1^ of electrical conductivity, respectively [22]. Above these thresholds, a 16% yield reduction is expected per each soil salinity unit increase [23]. In a saline environment, sorghum showed a certain ability to exclude Na [24] and limit Na transport from the roots to the leaves by unloading Na from the xylem into the roots [25,26,27,28]. Additionally, sorghum can compartmentalize Na into the cell vacuoles as an osmolyte to adjust osmosis at the cellular level and thereby compensate the potential drop in the growing medium [29]. Selective uptake and translocation of K and Ca versus Na were identified as further mechanism for salt tolerance in this plant [30]. However, above a certain threshold, Na can lead to a toxic accumulation in sorghum leaf and affect K, Ca, and Mg uptake and translocation [31,32], hampering photosynthetic activity and plant development [33,34]. The accumulation of osmoprotectants such as proline [35] and sugars [36], the increase in pigment levels (chlorophylls and carotenoids) [37], and the enhanced antioxidant enzymatic activity [38] are additional sorghum strategies to maintain cellular osmotic pressure, defend plant metabolism against reactive oxygen species (ROS), and protect the assembly of photosystems under salinity.

The goal of this study is to investigate sorghum response to the combined effects of soil and water salinity, and salt leaching through irrigation, on (i) plant growth and biometry (plant height, basal stem diameter, leaf number, final dry weight); (ii) leaf water relations (relative water content, water use efficiency, leaf water potential and its components); and (iii) ion (Na, K, Ca, and Mg) assimilation and allocation to plant organs. We expected leaching to reduce salt stress in sorghum, although plant acclimation processes to salinity and salt leaching are still not sufficiently known.

## 2. Materials and Methods

### 2.1. Acronyms

The acronyms used in this study are defined as follows: electrical conductivity of the saturation soil extract at 25 °C (EC_e_), electrical conductivity of water at 25 °C (EC_w_), leaching fraction (LF), salt leaching (SL), relative water content (RWC), root-to-shoot ratio (R:S), water use efficiency (WUE), dry weight (DW), fresh weight (FW), osmotic adjustment (OA), water potential (WP), osmotic potential (OP), turgor potential (TP), bioaccumulation factor (BAF), translocation index (TI), selective absorption (SA).

### 2.2. Experimental Set Up

The experiment was carried out in a greenhouse at the Department of Agricultural and Food Sciences (DISTAL), University of Bologna, Italy. *Sorghum bicolor* cv. Bulldozer (fiber sorghum) was grown for 103 days from 31 May to 11 September 2017. During this time, maximum and minimum air temperature and relative humidity remained consistently at 31.3 ± 3.1 °C, 25.5 ± 2.1 °C, and 52.9% ± 4.1%, respectively. 

The three factors, namely soil salinity (three levels), water salinity (three levels), and water regime (two levels), were cross-combined, resulting in 18 treatments (Table 1). Three completely randomized replicates were set up, totaling 54 pots. The 7 L pots were filled with sandy soil (80% sand, 13% silt, and 8% clay), previously sieved and mixed with table salt (NaCl) at 97% purity [39], to obtain the following treatments: control with no added salt (Ctrl), low soil salinity (LSS), and high soil salinity (HSS). LSS and HSS corresponded to electrical conductivity of the saturation soil extract at the standard temperature of 25 °C (EC_e_) [12] of 3 and 6 dS m^−1^, respectively. Soil EC_e_ in Ctrl was 0.27 dS m^−1^.

In the first two-thirds of the experiment (until August 8, i.e., 68 days after seeding), low water salinity (LWS) and high water salinity (HWS) were set at water electrical conductivity at the standard temperature of 25 °C (EC_w_) of 2 and 4 dS m^−1^, respectively; then, EC_w_ was increased to 4 and 8 dS m^−1^, respectively, until the end of the experiment. EC was measured with the benchtop CDM210 Conductivity Meter (Meter Lab). The amount of salt added to soil/water in order to reach the aforementioned salinity levels was calculated according to the following equation (1):

(1) $$\mathrm{TSS}\;\left(g_{NaCl}\;kg^{-1}\;\mathrm{soil}/\mathrm{water}\right)=\mathrm{ECe}\;\left(\mathrm{dS}\;m^{-1}\right)\times 0.640$$

NaCl concentration in tap water was 0.028 g L^−1^ (according to water supply company).

The pots were watered manually 2–3 times a week, determining the amount of water on a gravimetric basis. Two water regimes were imposed: one was maintaining pots close to field capacity while avoiding percolation and salt leaching (No SL), and the other was overirrigation to determine water drainage and, thereby, salt leaching (SL).

### 2.3. Plant Growth

Plant height, basal stem diameter. and leaf number were measured weekly. At harvest, shoots were divided into stems and leaves, and roots were recovered from the sandy soil. Root, stem, and leaf samples were oven-dried at 60 °C and weighed to determine the dry weight (DW) of the three plant organs and total plant biomass. The root-to-shoot ratio (R:S) was assessed on a DW basis.

### 2.4. Leaf Water Status

Leaf water potential (WP) (MPa) was assessed in the uppermost fully expanded leaf before harvest, through the WP4-C dewpoint potentiometer (METER Group, Pullman, WA, USA). The measurement was repeated after freezing and subsequently thawing the leaf to determine the osmotic potential (OP). Turgor potential (TP) was assessed as the difference between WP and OP.

The relative water content (RWC) (%) was determined on the same leaf. A small leaf disc of 2 cm diameter was cut from the leaf. It was weighed to determine fresh weight (FW) and was put in a 15 mL vial with distilled water in the dark. After 24 h, the turgid weight (TW) was measured, and then the sample was oven-dried at 105 °C for 24 h to assess the DW. The RWC (%) was calculated according to the following equation [40]:

(2) $$RWC=\frac{FW\;–\;DW}{TW\;–\;DW}\times 10$$

Leaf osmotic adjustment (OA) (MPa) was calculated according to the following formula [41]:

(3) $$\mathrm{OA}=({\mathrm{RWC}}_{C}\times {\mathrm{OP}}_{C})-\left({\mathrm{RWC}}_{\mathrm{ST}}\times {\;\mathrm{OP}}_{\mathrm{ST}}\right)$$

where RWC_C_ and RWC_ST_ indicate the RWC in the control and saline treatment, respectively, and OP_C_ and OP_ST_ indicate the OP in the control and saline treatment, respectively.

Water use efficiency (WUE) (kg DW L^−1^ H_2_O) was determined at harvest according to Equation (4) [42].

(4) $$\mathrm{WUE}=\frac{\mathrm{plant}\;\mathrm{DW}}{V_{H_{2}0}}$$

### 2.5. Mineral Elements

Dry samples of plant organs were ground, and the concentration of the main cationic elements (Na, K, Ca, and Mg) was quantified by inductively coupled plasma spectrometry (ICP-OES) (Spectro Arcos, Ametek, Kleve, Germany).

#### 2.5.1. Bioaccumulation Factor

The bioaccumulation factor (BAF) is defined as the ratio between the concentration of a given element in the plant (mg kg^−1^ DW) and its concentration in the soil (mg kg^−1^ soil DW). It was calculated for Na according to Equation (5):

(5) $$\mathrm{BAF}=\frac{C_{\mathrm{Na}}\;\mathrm{plant}\;\mathrm{tissue}}{C_{\mathrm{Na}}\;\mathrm{soil}}$$

where C_Na_ is Na concentration (mg kg^−1^ DW). Greater BAF values indicate lower ion retention in soil colloids or higher root ability to extract ions [43].

#### 2.5.2. Selective Absorption

The selective absorption (SA) of K and Ca quantifies the root ability to adsorb K and Ca over Na and is calculated according to Equation (6):

(6) SA(Ca1)=NaCa1 soilNaCa1 plant×100

where the superscript ^1^ refers to the concentration of Ca, K or Mg (g kg^−1^ DW). Higher SA values indicate stronger exclusion of Na^+^ and selective absorption of Ca, K or Mg by the roots [44]. 

#### 2.5.3. Translocation Index

The translocation index (TI) is defined as the ratio between the content (element concentration × DW) of a given element in a plant organ and the content in the whole plant. The TI was calculated to quantify element partitioning to roots (TI_R_), stem (TI_S_), and leaves (TI_L_) according to the following equations [45]:

(7) $${\mathrm{TI}}_{R}=\frac{C_{Na^{1}}\;\mathrm{roots}}{C_{Na^{1}\;}\mathrm{roots}\;+{\;C}_{Na^{1\;}}\mathrm{stem}\;+{\;C}_{Na^{1\;}}\mathrm{leaves}}\times 100$$

(8) $${\mathrm{TI}}_{S}=\frac{C_{Na^{1}}\;\mathrm{stem}}{C_{Na^{1\;}}\mathrm{roots}\;+{\;C}_{Na^{1}}\;\mathrm{stem}\;+{\;C}_{Na^{1}}\mathrm{leaves}}\times 100$$

(9) $${\mathrm{TI}}_{L}=\frac{C_{Na^{1}}\;\mathrm{leaves}}{C_{Na^{1}}\;\mathrm{roots}\;+{\;C}_{Na^{1}}\;\mathrm{stem}\;+{\;C}_{Na^{1\;}}\mathrm{leaves}}\times 100$$

where C is the ion content (mg) in the specific plant organ and the superscript ^1^ refers to Na, Ca, K or Mg.

#### 2.5.4. Vector Analysis of Dry Weight and Element Concentration and Content

The dynamics of the aforementioned elements in the plant’s tissues triggered by the saline treatments were represented through a vector analysis diagram [46]. This system shows the simultaneous changes in total plant biomass (DW) and element concentration and content in the plants exposed to salinity. DW and element concentration and content were expressed as relative data with respect to the Ctrl No SL, which was set at 100%. The three-dimensional vector analysis diagram has the element content on the horizontal axis and the element concentration on the vertical axis, while DW intervals are plotted as diagonal axes. The observation of the three parameters’ shifts in a single graph facilitates the assessment of each element’s status, i.e., element dilution, deficiency, sufficiency, luxury uptake, toxicity, and multielement interactions [46].

### 2.6. Statistical Analysis and Data Presentation

To better highlight the key effects of the experiment, we report data from the four corner treatments, i.e., those encompassing the full range of the three factors’ levels (Table 1): Ctrl No SL (Control + No Salt Leaching), HSS-HWS No SL (High Soil Salinity + High Water Salinity + No Salt Leaching), Ctrl SL (Control + Salt Leaching), and HSS-HWS SL (High Soil Salinity + High Water Salinity + Salt Leaching).

Data of plant growth, leaf water status, and mineral elements were analyzed in a one-way analysis of variance (ANOVA) using the CoStat 6.4 package (CoHort Software, Berkeley, CA, USA). Prior to statistical analyses, all data were tested for homogeneity of variance through the Bartlett test. Wherever necessary, data were log-transformed to ensure homogeneity of variance. The LSD test at *p ≤* 0.05 was used to indicate significant differences among treatments.

Data of final plant morphology, growth, and leaf water traits in the 18 original treatments and the *p*-levels in the three-way ANOVA are reported in Table S1 of the Supplementary Materials.

A principal component analysis (PCA) was performed with JMP 15 (SAS Institute Inc., Cary, NC, USA) on biomass (DW), morphological (PH, SD, LF, R:S) and leaf water traits (WUE, RWC, WP, OP, TP), and element accumulation indices (BAF, SA, TI) to reduce the number of variables into a smaller number of principal components accounting for most of variance in the original dataset.

## 3. Results

### 3.1. Water and Na Input to the System

The total amounts of water and Na supplied during the experiment are reported in Table 2. The two treatments Ctrl SL and HSS-HWS SL received +63% and +100% more water, respectively, than the corresponding No SL treatments. The water outputs, i.e., the amount percolated, were negligible under No SL, while they amounted to 15.9–21.5 L under SL. The leaching fraction (LF), i.e., the amount of water lost in percent of the amount supplied, was 28.8% and 43.8% in Ctrl SL and HSS-HWS SL, respectively.

The total Na input with soil and water (Table 2) was negligible in the two Ctrl groups (~4 g pot^−1^), whereas it reached 25.2 and 43.2 g pot^−1^ in HSS-HWS No SL and SL, respectively. In HSS-HWS No SL, the soil Na input was higher that the water Na input; the opposite occurred in HSS-HWS SL. The loss of Na through leaching was negligible in the Ctrl under both No SL and SL; it was small in HSS-HWS No SL, and it was relevant in HSS-HWS SL. Based on Na input (soil and water) and output (leaching), the amount of Na remaining in the system at the end of the experiment was an average 3.9 g in the two Ctrl groups (No SL and SL) and an average 25.3 g in the two HSS-HWS treatments (No SL and SL).

### 3.2. Morphological Traits

**Figure 1:**
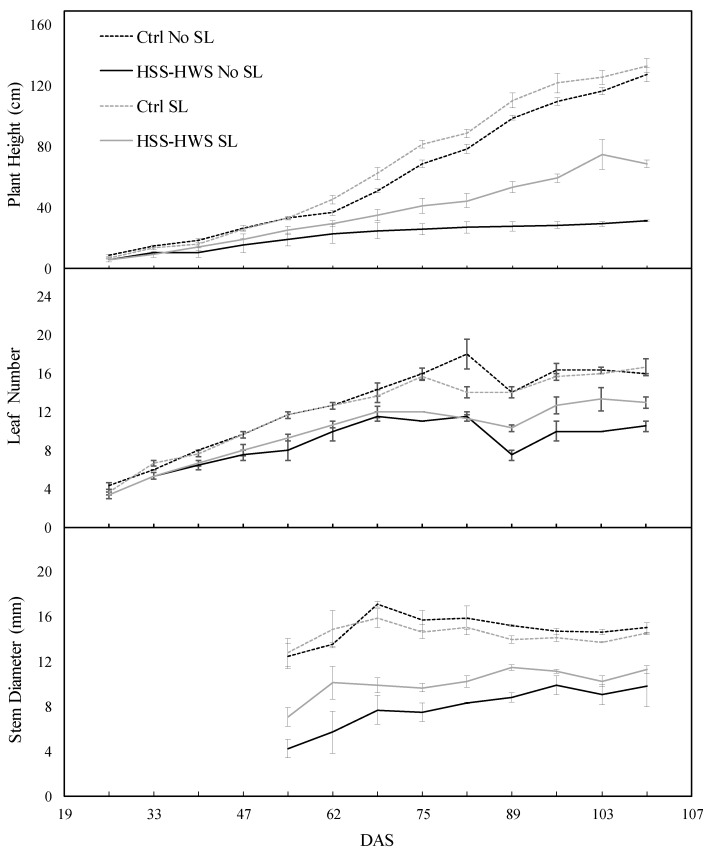
Time trend of sorghum morphological traits in four treatments at variable soil and water salinity and salt leaching. Ctrl, control; HSS-HWS, high soil salinity and high water salinity; SL, salt leaching; DAS, days after seeding. Vertical bars indicate ± standard error (n = 3).

Soil and water salinity determined stunted growth resulting in a reduced plant height, number of leaves, and stem diameter (Figure 1). However, under HSS-HWS, SL had a mitigating effect on plant height and leaf number, compared to No SL. On the contrary, in the Ctrl, the extra amount of water supplied with SL did not significantly determine higher measures of morphological traits; this indicates that the amount of water supplied with No SL was nonlimiting for plant growth. 

At the end of the experiment, plant height was reduced by 47% and 76% under HSS-HWS SL and HSS-HWS No SL compared to the averaged Ctrl SL and No SL, respectively. Leaf number decreased by 30% and 23% under HSS-HWS SL and HSS-HWS No SL, respectively. Stem diameter decreased by 35% and 20% under HSS-HWS SL and HSS-HWS No SL, respectively.

### 3.3. Final Plant Growth

**Figure 2:**
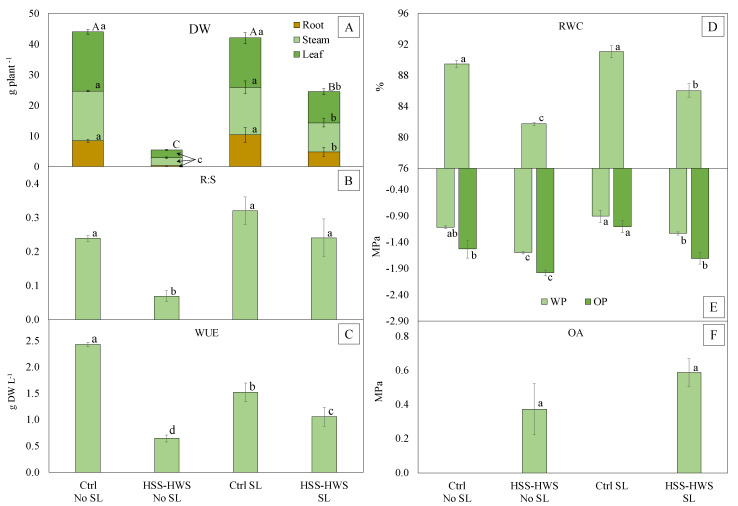
(**A**) Dry weight (DW); (**B**) root-to-shoot ratio (R:S); (**C**) water use efficiency (WUE); (**D**) relative water content (RWC); (**E**) water and osmotic potential (WP and OP, respectively); and (**F**) osmotic adjustment (OA) in the four treatments. Ctrl, control; HSS-HWS, high soil salinity and high water salinity; SL, salt leaching. Vertical bars indicate ± standard error (n = 3). Different letters indicate significant differences at *p ≤* 0.05. In Figure 1A (DW), lowercase and uppercase letters indicate statistical differences (*p ≤* 0.05) among treatments in single organs and their totals, respectively.

Soil and water salinity had a negative effect on plant biomass, root-to-shoot ratio, and WUE. HSS-HWS had a stronger impact on final dry weight under No SL than SL (Figure 2A), in accordance with morphological traits (Figure 1). Under control condition, final dry weight was comparable between SL and No SL, indicating that the amount of water distributed in this latter treatment was nonlimiting for plant growth. The root-to-shoot ratio was significantly lower only in HSS-HWS No SL, while that of HSS-HWS SL was comparable with the two Ctrl groups (Figure 2B). WUE was greatest in the Ctrl No SL, followed by the Ctrl SL (Figure 2C). This was due to similar plant biomass in these two treatments (Figure 2A), in contrast to a higher amount of water used by Ctrl SL (Table 1). WUE dropped more heavily under HSS-HWS with No SL than SL (Figure 2C). This was due to a stronger reduction in plant biomass (Figure 2A) than in the amount of water used (Table 1) under No SL.

### 3.4. Leaf Water Status

Soil and water salinity affected leaf water status. RWC was lower in HSS-HWS, and the decrease was stronger under No SL than SL (Figure 2D). WP and OP also decreased due to HSS-HWS (Figure 2E). However, under No SL both WP and OP were more negative than under SL. A certain decrease in the two potentials was also registered in the Ctrl No SL. The turgor potential (TP) (not shown) was not significantly affected by soil salinity, water salinity, or SL, likely because of osmotic adjustment (OA). However, OA did not vary significantly in the two salinity treatments (Figure 2F). 

### 3.5. Cation Accumulation and Translocation

High soil and water salinity (HSS-HWS) determined sizeable increases in Na concentration in roots, stem, and leaves with respect to nonsaline Ctrl, both under No SL and SL (Table 3). HSS-HWS No SL also showed a higher leaf Na concentration than HSS-HWS SL. Potassium concentration counter-balanced Na concentration, but only at root level (Table 3): in fact, higher K_(R)_ concentrations were found in the two Ctrl groups (average 7.60 mg kg^−1^) vs. the two HSS-HWS (average 3.99 mg kg^−1^). Lastly, Ca and Mg concentrations were influenced by salinity only at shoot, i.e., stem and leaf, level (Table 3): the concentration of these two elements decreased in both organs under salinity; however, HSS-HWS No SL suffered a stronger decrease than HSS-HWS SL.

Sodium bioaccumulation peaked in HSS-HWS No SL (BAF 3.16), compared to the other three treatments, which were statistically similar (average BAF 1.67) (Table 3). This indicates that Na plant concentration significantly increased with respect to Na soil concentration only under No SL.

Selective absorption of K was mildly (*p* ≤ 0.10) reduced by salinity (Table 3), indicating loss of plant ability to select this macronutrient under Na-enriched environment. The same pattern was shown for SA_(Mg)_ and, only in HSS-HWS No SL, for SA_(Ca)_.

**Figure 3:**
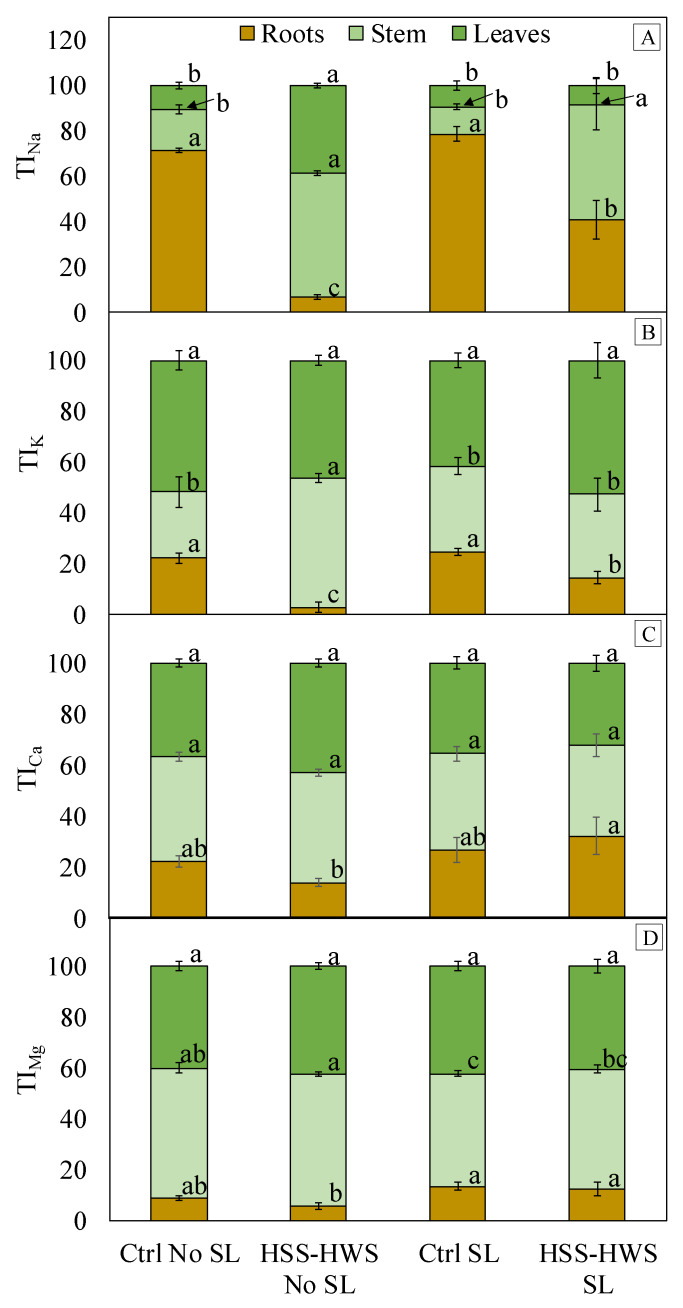
Translocation Index (TI) of (**A**) sodium, (**B**) potassium, (**C**) calcium, and (**D**) magnesium to the roots, stem, and leaves. Ctrl, control; HSS-HWS, high soil salinity and high water salinity; SL, salt leaching. Vertical bars indicate ± standard error (n = 3). Different letters indicate significant differences among treatments at *p ≤* 0.05.

Translocation indices address the relationships in element contents (Figure 3), while the above-described BAF and SA relate to relationships in element concentrations. More than 50% of the amount of Na taken up by the plant remained in the roots under no salinity, whereas the saline environment (HSS-HWS) determined an upsurge of this element to stem and leaves (TI_Na,_ Figure 3A). This was especially true in HSS-HWS No SL, where leaves, which are the most delicate of the three plant organs, received almost 40% of the total amount of Na. The strongest differences in TI_K_ among treatments concerned roots (Figure 3B). HSS HWS No SL had a similar effect on TI_K_ in the three organs as it did on TI_Na_ (Figure 3A). The other three treatments allocated more K to leaves. Small differences among treatments were observed for TI_Ca_ and TI_Mg_ (Figure 3C,D, respectively): for both elements, the stem was more important than roots for the allocation of these elements.

**Figure 4:**
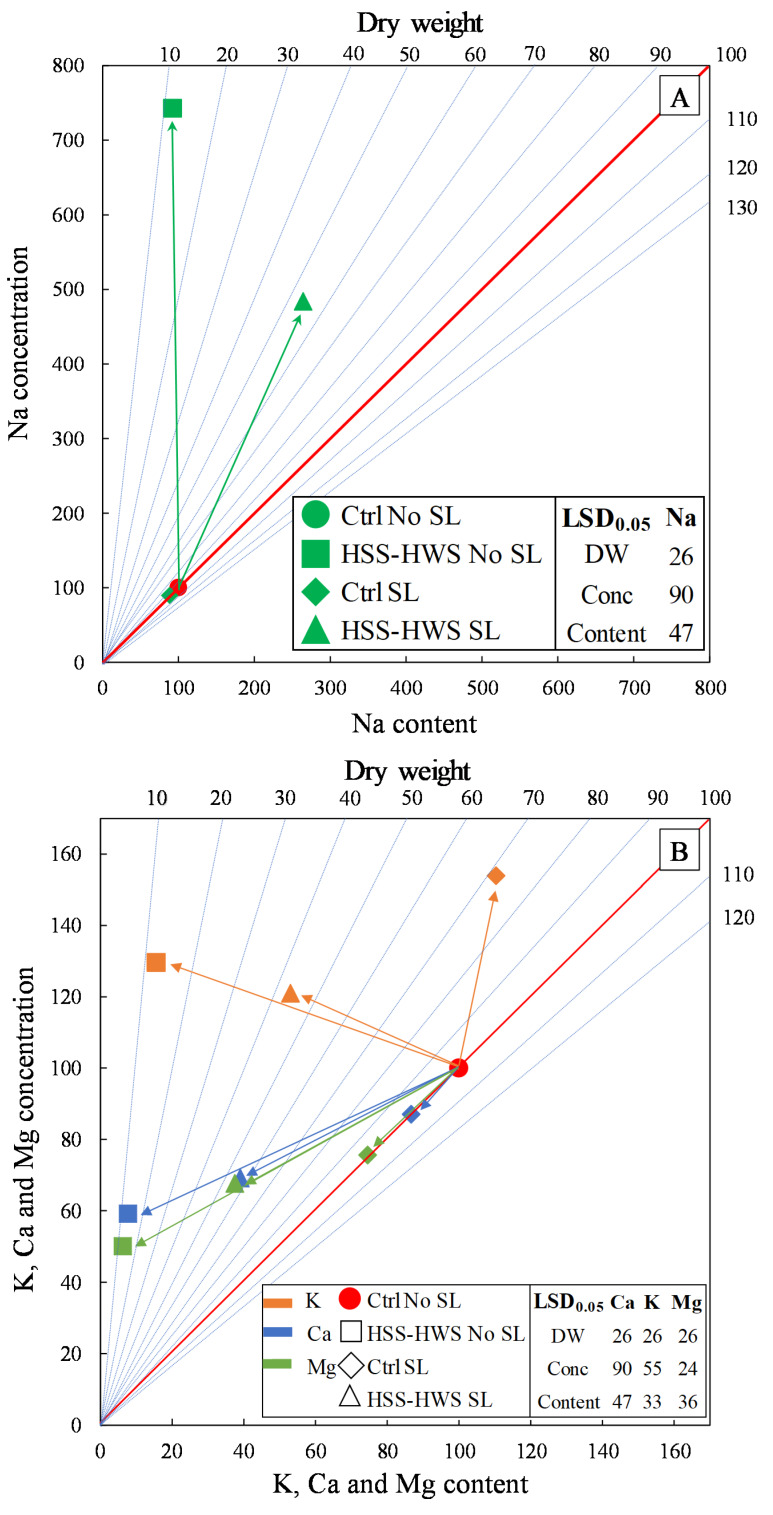
(**A**) Vector analysis showing directional changes in biomass and Na content and concentration in sorghum plants and, (**B**) vector analysis showing directional changes in relative biomass and K, Ca, and Mg content and concentration in sorghum plants. Dry weight and element content and concentration are expressed as relative data with respect to the Ctrl No SL treatment, which is set at 100% and is indicated by a red filled circle.

The vector analysis combines changes in biomass (Figure 2A) and element concentration (Table 3) and content (Figure 3) into a comprehensive picture of plant response to Na input (Figure 4). Overall, the strongest variations were associated with Na, the element that we directly supplied in soil and water (Figure 4A). However, changes were also observed for K, Ca, and Mg (Figure 4B).

High salinity without leaching (HSS-HWS No SL) determined Na toxicity, i.e., strong increase in Na concentration and concurrent drop in biomass, resulting in approximately the same Na content in the whole plant as found in the nonsaline reference treatment (Ctrl No SL) (Table 4). K and Ca were not influenced in terms of concentration, whereas their content decreased proportionally with biomass reduction. In the Ctrl SL, the extra amount of water resulted in water excess, i.e., no biomass increase and no changes in element concentration and, therefore, content. Lastly, high salinity with salt leaching (HSS-HWS SL), involving extra amounts of both water and salt with respect to HSS-HWS No SL (Table 1), determined Na and water excess, i.e., biomass reduction and a more than proportional increase in Na concentration, resulting in higher Na content. In contrast to Na, the other elements had the same response as in the HSS-HWS No SL treatment.

### 3.6. Principal Component Analysis of Plant Traits

The PCA of biomass, leaf physiological traits, and element concentrations in sorghum organs extracted two main principal components (eigenvalues and loadings in Tables S2 and S3, respectively), constituting 72.4 % of the total variation.

**Figure 5:**
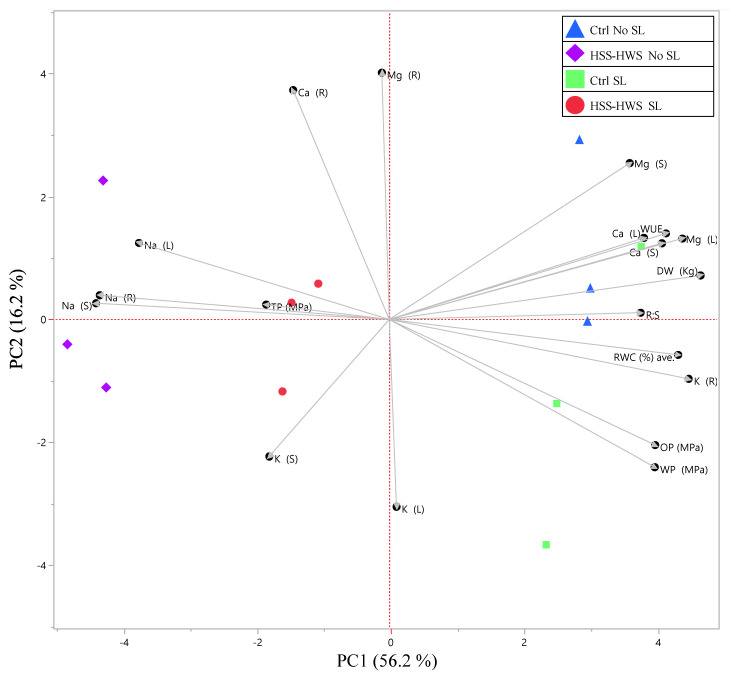
Biplot of the principal component analysis of biomass, morphological and leaf water status traits, and element translocation indices in the four treatments. The amount of variation associated with each PC is indicated in brackets. Ctrl, control; HSS-HWS, high soil salinity and high water salinity; SL, salt leaching; Na(R), Na(S), and Na(L), Na concentration in roots, stem, and leaves, respectively; K, Ca, and Mg followed by (R), (S), and (L) indicates K, Ca, and Mg concentrations in the respective organs; DW, plant dry weight; R:S, root to shoot ratio; RWC, relative water content; WP, leaf water potential; OP, osmotic potential; TP, turgor potential.

The biplot of PC1 and PC2 (Figure 5) showed that they contributed to 56.2% and 16.2 % of the total variation, respectively. It showed a net separation between the Ctrl groups (blue triangles and green squares), placed in the positive side of PC1 axis, and the HSS-HWS treatments (red dots and purple rhombuses), placed in the negative side of PC1 axis.

The PC1 had high negative loadings for Na_(R)_, Na_(S)_, Na_(L)_, and TP and positive loadings for R:S ratio, meaning that these parameters separated HSS-HWS No SL from SL and separated these two treatments from the controls. Hence, it is sensed that PC1 represents the effects of salinity on plant growth.

PC2 separated SL from No SL treatments, although the separation was imperfect. The parameters that accounted for PC2 are Ca_(R)_ and Mg_(R)_, which had high positive loadings. This is consistent with the ANOVA of Ca and Mg translocation indices (TI) to the roots, which were higher in HSS-HWS SL than in HSS-HWS No SL (Figure 3C,D). The analysis also identified K_(L)_ and K_(S)_ as important components of PC2, with high negative loadings on PC2. Potassium concentration (Table 2) and translocation (Figure 3) to the leaves were not statistically different between HSS-HWS SL and No SL. Notably, K concentration and translocation to the roots decreased significantly under HSS-HWS SL and, to a greater extent, under No SL. Potassium translocation to the stem increased significantly only in HSS-HWS No SL, although K concentration resulted as comparable with the other three treatments. This circumstance and the weak loadings of K_(S)_ and K_(L)_ on the PC1 (salinity) suggest that salinity mainly alters K homeostasis at the root level, whereas K status in the stem and leaves depends more on SL.

## 4. Discussion

Under high soil and water salinity (HSS-HWS), sorghum incurred a significant reduction in plant height and leaf number, as observed in a previous study [47]. These effects were stronger in No SL: in fact, SL introduced 71% more Na into the system (Table 2) but promoted Na leaching from the soil profile. This resulted in only a 15% higher amount of residual Na in the soil–plant system at the end of the experiment.

In saline soils, leaching decreases the osmotic potential of soil solution, consequently increasing soil moisture and permanent wilting point [22]. High soil moisture prevents plant wilting, so a higher irrigation volume is a strategy to increase water availability with saline water. On the contrary, under no salt leaching, water uptake and plant water status are rapidly impaired by salinity. Salt stress delays cell division and elongation [48], affecting foliar differentiation, expansion, and internode length, while concurrently accelerating leaf senescence [49,50]. This explains why in our study stem elongation and leaf development were less severely affected by salinity under SL than No SL, despite the higher amount of salt supplied with SL (Table 2).

In contrast to our results, Joardar et al. [34] did not observe any change in sorghum height, leaf number, and stem diameter with EC_w_ up to 7.18 dS m^−1^, while Jafari et al. [51] observed a significant reduction in plant height but not leaf number at 80 mM EC_w_ (≈ 8 dS m^−1^). The inconsistency between our study and these sources may at least partially be due to differential genotype tolerance within the *Sorghum bicolor* species [52,53] Additionally, in our study the combined effect of soil and water salinity could lead to a stronger impact than that of water salinity alone in the cited sources.

The stunted plant growth determined by salinity resulted in lower DW and R:S (Figure 2), as observed by several sources [31,53,54,55]. Biomass reduction may be due to increased respiration in response to salt stress [56] or due to toxic ion accumulation [57], while the higher release of ethylene under stress may have inhibited root and shoot growth and decreased their ratio [58]. The R:S decrease under salinity indicates plant reaction to reduce root exposure to the hostile environment. This is in contrast to drought stress that drives plants to expand their root system to explore a larger soil volume in search of water [59]. However, this hypothesis is not supported by the findings of Jafari et al. [51], De Lacerda et al. [60], and Al-Amoudi and Rashed [61], who reported a sorghum R:S increase at salinities up to 90, 100, and 240 mM NaCl, respectively, corresponding to 9, 10, and 24 dS m^−1^ of EC_w_. Aishah et al. [62], instead, found a sorghum R:S increase up to 10 dS m^−1^ of EC_w_, followed by a drop at higher values. Lastly, Mahmood et al. [63] did not observe any sorghum R:S change up to 24 dS m^−1^. According to Shannon et al [64], a R:S increase under salinity is the premise for a better use of soil moisture and nutrients. Comparing our study with these sources where higher EC_w_ levels were tested, it is sensed that the plant only allocates more resources to the root system above a certain salinity threshold, as a mechanism to escape salt stress. Conversely, at salinity levels similar to our case, the plant reacts by reducing its root biomass to minimize salt exposure and control Na uptake [51]. Moreover, the R:S decrease may indicate a stronger carbon allocation to the photosynthetic organs in order to increase carbon assimilation, as mechanism of acclimation to salt stress [65].

Guimarães et al. [66] found a 50% WUE decline with an EC_w_ of 6.9 dS m^−1^ in sorghum, in accordance with the sharp WUE decrease observed in our experiment (Figure 3D). Richardson and McCree [67] and Yan et al. [68] argued that sorghum reduces stomatal conductance and transpiration under salinity, potentially leading to WUE increases. However, reduced stomatal conductance limits photosynthesis and final biomass. Reduced biomass was the main cause for WUE loss in our study.

The decrease of leaf RWC, WP, and OP under salinity (Figure 2D,E) reflects the findings of Netondo et al. [31], who obtained similar results in sorghum at EC_w_ up to 25 dS m^−1^. However, the strongest drop in that study was observed between 0 and 100 mM NaCl.

In our study, the strongest reduction in RWC was observed in No SL, although less salt was supplied compared to SL (Table 2). A decrease in RWC is normally associated with turgor loss, because of limited water availability [69]. In our study, the plant was able to adjust osmotically (Figure 2F) and maintain leaf water balance and turgor potential. However, plant growth was seriously impaired by salt stress. In salt-exposed plants, the cations supplied with saline water (Ca, K, Na) play a key role in OA [70], as their uptake and use in plant tissues are less energy consuming than the production of organic solutes to be used as osmoregulating compounds for OA [63,64]. The OP reduction observed in salt-treated plants (Figure 2E) is the likely consequence of intracellular accumulation of osmoregulating compounds and cations, which is a key mechanism, together with intercellular compartmentalization, to perform OA [71]. Lower OP values generally indicate higher OA and water retention in plant tissues [72]. In our experiment, the higher water volume supplied with HSS-HWS SL did not determine a higher OA (i.e., stronger OP reduction) compared to HSS-HWS No SL. However, both OP and RWC benefitted from higher water supply (SL), as they decreased less than under No SL. Therefore, it is evinced that with higher soil moisture and SL, the plants were less hampered by salt stress and necessitated a smaller OA to cope with the stress.

Sodium was the cation that accumulated most steeply in sorghum plants under salinity (Figure 4A). Na accumulation in roots is a tolerance strategy: the consequent reduction in root OP can sustain water uptake. Conversely, the leaf accumulation of potentially toxic Na could slow down, or even stop, photosynthesis [73]. Our results suggest that under salinity sorghum roots were saturated with Na, forcing Na translocation to the stem. When salinity (HSS-HWS) was associated with No SL, Na reached stem saturation and a significant amount of Na was translocated to the leaves (Figure 4A). De Lacerda et al. [74] observed a similar increase in Na allocation to the shoot in sorghum genotypes irrigated with water at 100 mM NaCl (10 dS m^−1^). This was in contrast to Niu et al. [54], who did not observe changes in Na uptake in sorghum genotypes irrigated with water at 8 dS m^−1^ EC_w_.

The maintenance of low cytosolic Na concentration and Ca/Na and K/Na homeostasis is another mechanism of salt tolerance [75]. Ca plays a key role in the response to abiotic stress, acting as second messenger in the pathway of stress signal transduction [76,77]; it also acts in exocytosis [77] to exclude toxic ions. K is involved in turgor control: inhibition of K uptake leads to stunted growth [73]. The K and Na ions have similar radius and hydration energy [78] and can be taken up jointly under sodic conditions. K/Na selective absorption depends on cell wall and plasma membrane (PM) integrity. Salinity promotes Ca accumulation at the root level in order to increase Na exclusion and preserve K accumulation [79]. However, highly concentrated Na can displace Ca in the cell wall fibrils and PM binding sites, causing membrane depolarization and cell wall instability [80]. The resulting K/Na imbalance prompts uncontrolled Na influx and cytosolic K leakage from the cell [81]. Indeed, higher doses of Ca, K, and Mg under salinity help the plant to contrast nutrient imbalance [61]. 

Higher water availability in HSS-HWS SL maintained SA_(Ca)_ at the same level as nonsaline Ctrl (Table 3), while increasing Ca allocation at the root level (Figure 3C). Under HSS-HWS, the higher Ca accumulation in roots vs. shoots may indicate the plant’s attempt to maintain selective transport across membranes. Furthermore, restricted root growth can limit Ca uptake and transport from root to shoot, in spite of the transpiration stream [82]. Inhibition of Ca flux in the phloem was also observed under salinity [31], causing leaf deficiency and reduced photosynthetic rate.

As it concerns K, restrained allocation to shoots was observed in sorghum under salinity [32,57], which was in contrast to no change found in sorghum K uptake and allocation to the upper organs in another study [65] and also in contrast to increased leaf K concentration with salinity [53]. In our study, K concentration and translocation decreased only in the roots under salinity (Table 3, Figure 3B), reaching the lowest value in No SL. It may be assumed that K was more translocated to the shoot in order to maintain high K concentration in the leaves, where this ion plays a key role in maintaining leaf turgor. However, the PC2 showed that leaf K concentration was related to a combined effect of salinity, water availability, and SL, rather than salinity alone (Figure 5). 

Additionally, the ability to selectively adsorb K and Ca over Na is not sufficient to assure cation homeostasis. In fact, Na can also be transported through the apoplastic transpiration stream, bypassing all filter barriers imposed by cell membranes [83].

The reduction in plant Mg content was proportional to biomass reduction (Figure 4B). Mg root allocation was significantly lower in HSS-HWS No SL. However, Mg translocation to the stem was not related to salinity, as it was lower in SL treatments with both saline and nonsaline water (Figure 3D). Despite unaltered Mg translocation to the leaves (Figure 3D), Mg leaf concentration declined dramatically with HSS-HWS (Table 3), supporting the findings by Netondo et al. [31] who reported a constraint in Mg leaf incorporation under salinity.

## 5. Conclusions

The present study demonstrates that salt leaching, although performed with saline water, alleviates salt stress in *S. bicolor* by reducing the detrimental effects exerted by salinity on plant growth, leaf water status, and cation homeostasis across plant organs.

Sodium input to the soil and irrigation water resulted in higher Na concentration in plant organs. This was especially true in the case of no salt leaching. Under this circumstance, the plant had to deploy a special effort to maintain cation (K, Ca, and Mg) homeostasis and counter Na upsurge from the root apparatus to the leaves. The stem appeared to act as a buffer organ, trying to maintain cation balance and prevent Na from reaching the delicate photosynthetic organs.

In the frame of soil and water salinity, a higher irrigation volume determined a higher salt input to the system but nevertheless is able to mitigate the noxious effects associated with Na accumulation in plant organs. In contrast to this, conservative irrigation, i.e., limiting salt input to the system by avoiding the extra supply of water needed for salt leaching, was proved to be a losing strategy that worsened Na effects by hampering plant water uptake and cation selective absorption. 

Although the practice of salt leaching when using saline water leads to a more tolerable rhizosphere environment, further research is needed to evaluate the long-term sustainability of this method, assess Na fate in the soil–plant system, and investigate Na impact on soils and aquifers.

## Figures and Tables

**Table 1:** Scheme of the 18 treatments obtained by combining three levels of soil salinity (EC_e_), three levels of water salinity (EC_w_), and two water regimes (SL). Shaded rows indicate the four corner treatments whose data are discussed in this paper.

Treatment No.	Soil Salinity EC_e_ (dS m^−1^)	Water Salinity EC_w_ (dS m^−1^)	Salt Leaching (SL)
1	0	0	No
2	0	2–4	No
3	0	4–8	No
4	3	0	No
5	3	2–4	No
6	3	4–8	No
7	6	0	No
8	6	2–4	No
9	6	4–8	No
10	0	0	Yes
11	0	2–4	Yes
12	0	4–8	Yes
13	3	0	Yes
14	3	2–4	Yes
15	3	4–8	Yes
16	6	0	Yes
17	6	2–4	Yes
18	6	4–8	Yes

**Table 2:** Total amount of water and Na supplied to the system.

Treatment	Water Input (L)	Water Output (L)	LF (%)	Na Input Soil (g)	Na Input Water (g)	Na Output with Leaching (g)	Na Output–Input (g)
**Ctrl** **No SL**	33.8	0.8	2.4	3.6	0.4	0.02	4.0
**HSS-** **HWS** **No SL**	24.6	0.5	2.1	15.2	10.0	1.59	23.6
**Ctrl** **SL**	55.1	15.9	28.8	3.6	0.6	0.33	3.8
**HSS-** **HWS** **SL**	49.1	21.5	43.8	15.2	27.9	16.09	27.1

LF, leaching fraction; Ctrl, control; HSS-HWS, high soil salinity and high water salinity; SL, salt leaching.

**Table 3:** Na, K, Ca, and Mg concentrations (mg kg^−1^ DW) in roots (R), stem (S), and leaves (L); Na bioaccumulation factor (BAF); and K, Ca, and Mg selective absorption (SA). Different letters indicate significant differences at *p* ≤ 0.05.

Treatment	Na_(R)_	Na_(S)_	Na_(L)_	K_(R)_	K_(S)_	K_(L)_	Ca_(R)_	Ca_(S)_	Ca_(L)_	Mg_(R)_	Mg_(S)_	Mg_(L)_	BAF_(Na)_	SA_(K)_	SA_(Ca)_	SA_(Mg)_
**Ctrl** **No SL**	3.19 b	0.44 b	0.20 c	7.39 a	4.76	7.62	8.62	8.31 a	6.25 a	1.68	5.20 a	3.40 a	1.72 b	0.79	0.07 a	0.31 a
**HSS-** **HWS** **No SL**	8.66 a	8.47 a	6.59 a	3.32 c	8.11	7.83	9.74	3.89 c	4.19 b	1.69	2.08 b	1.70 c	3.16 a	0.51	0.02 b	0.08 b
**Ctrl** **SL**	2.50 b	0.25 b	0.18 c	7.82 a	6.97	8.08	7.39	6.48 b	5.89 a	1.56	3.39 b	3.08 ab	1.58 b	1.04	0.06 a	0.26 a
**HSS-** **HWS** **SL**	8.58 a	5.99 a	0.71 b	4.66 b	5.73	7.58	8.62	4.66 bc	3.91 b	1.61	3.06 b	2.44 bc	1.73 b	0.73	0.05 a	0.23 b
***p***	0.001 **	0.001 **	0.001 **	0.001 **	0.338 ns	0.833 ns	0.651 ns	0.022 **	0.018 *	0.962 ns	0.080 **	0.0043 **	0.018 *	0.054 ^(+)^	0.003 **	0.002 **

Ctrl, control; HSS-HWS, high soil salinity and high-water salinity; SL, salt leaching. ns, (+), *, and ** mean not significant and significant at *p* ≤ 0.10, *p* ≤ 0.05, and *p* ≤ 0.01, respectively.

**Table 4:** Interpretation of the directional changes in relative dry weight (DW) and element concentration and content with respect to the reference treatment (Ctrl No SL), shown in Figure 4. Upwards and downwards arrows indicate significant changes, and (**~**) indicates insignificant changes. The three increasing arrow slopes indicate an increasing amplitude of the variation (from >1 LSD to >3 LSD).

Treat.	DW	Elem.	Conc.	Cont.	Interpretation
**HSS-HWS No SL**	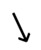	Na	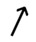	~	**Na toxicity** Excess Na associated with normal soil moisture caused a strong decrease in biomass and K, Ca, and Mg content. However, the concentration of all these elements except Mg remained constant, meaning that the reduction in their content was proportional to biomass reduction. The drop in Mg concentration indicates a reduction in Mg uptake proportionally greater than biomass reduction. Na content remained unvaried but, due to the drastic biomass reduction, its concentration increased dramatically.
		K	~	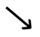	
		Ca	~	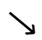	
		Mg	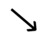	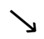	
**CTRL SL**	~	Na	~	~	**Water excess** Water availability exceeding the soil water holding capacity did not determine extra biomass gain, nor did it influence Na, K, Ca, and Mg concentration and content.
		K	~	~	
		Ca	~	~	
		Mg	~	~	
**HSS-HWS SL**	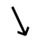	Na	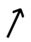	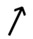	**Na and water excess** Irrigation with saline water exceeding the soil water holding capacity slightly reduced K, Ca, and Mg concentration and content and plant biomass. The concentration of all these elements except Mg remained constant, meaning that the reduction in their content was proportional to biomass reduction. The drop in Mg concentration indicates a reduction in Mg uptake proportionally greater than biomass reduction. Na concentration and content, on the contrary, increased considerably.
		K	~	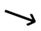	
		Ca	~	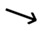	
		Mg	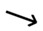	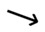	

## Supplementary Materials

The following are available online at https://www.mdpi.com/2223-7747/9/5/561/s1, Table S1: Final plant morphology, growth and leaf water traits in the 18 treatments obtained by combining three levels of soil salinity (EC_e_), three levels of water salinity (EC_w_) and two water regimes (SL), and statistical significance of the three simple factors and their interactions, Table S2: Eigen analysis of the correlation matrix, Table S3: Factorial loadings for principal component.
