# Supplementary material for: Salt Tolerance and Na Allocation in Sorghum bicolor under Variable Soil and Water Salinity
Source: Plants (Basel). 2020 Apr 28;9(5):561. doi: 10.3390/plants9050561 (PMC7284944; doi:10.3390/plants9050561)
Supplement: Supplementary file 1 [file plants-09-00561-s001.pdf]

Table S1: Final plant morphology, growth and leaf water traits in the 18 treatments obtained by combining three levels of soil salinity (EC_e), three levels of water salinity (EC_w) and two water regimes (SL), and statistical significance of the three simple factors and their interactions.

Treatment no.	EC_e (dS m^{-1})	EC_w (dS m^{-1})	SL	PH (cm)	LN	SD (mm)	DW (g $plant^{-1}$)	R:S	WUE (g L^{-1})	RWC (%)	WP (MPa)	OP (MPa)	OA (MPa)
1	0	0	No	127.3	15.0	16.0	44.1	0.24	2.4	89.4	-1.11	-1.32	
2	0	2 - 4	No	94.7	13.7	16.0	44.7	0.21	3.0	87.5	-1.25	-1.54	0.69
3	0	4 - 8	No	55.0	11.0	13.0	27.5	0.15	1.5	88.8	-1.18	-1.48	0.56
4	3	0	No	84.5	14.0	14.0	42.3	0.21	2.7	87.0	-1.28	-1.56	0.61
5	3	2 - 4	No	51.2	12.1	13.3	22.9	0.13	1.9	85.7	-1.62	-2.22	2.91
6	3	4 - 8	No	30.0	11.5	11.0	6.6	0.09	0.8	82.4	-1.71	-2.08	1.67
7	6	0	No	57.5	13.3	13.0	27.0	0.10	2.2	82.6	-1.72	-2.07	1.79
8	6	2 - 4	No	36.7	12.3	10.0	11.8	0.14	1.1	81.6	-1.75	-1.98	0.24
9	6	4 - 8	No	31.5	9.8	10.5	5.5	0.07	0.6	83.0	-1.65	-2.01	1.49
10	0	0	Yes	133.2	14.5	16.7	42.0	0.32	1.5	90.7	-0.99	-1.01	
11	0	2 - 4	Yes	130.2	13.3	15.0	30.8	0.30	1.3	90.2	-1.02	-1.52	1.85
12	0	4 - 8	Yes	108.8	14.2	15.3	30.5	0.31	1.2	88.3	-1.07	-1.59	1.99
13	3	0	Yes	93.3	11.6	14.7	28.9	0.21	1.2	87.8	-1.19	-1.42	1.33
14	3	2 - 4	Yes	90.3	11.3	15.0	31.8	0.25	1.3	87.2	-1.16	-2.02	3.37
15	3	4 - 8	Yes	76.2	10.7	13.3	30.3	0.23	1.3	86.0	-1.58	-1.77	2.43
16	6	0	Yes	85.8	13.4	14.3	36.8	0.17	1.5	82.8	-1.48	-1.99	2.90
17	6	2 - 4	Yes	78.3	11.3	13.7	22.6	0.16	1.0	88.2	-1.39	-1.88	3.00
18	6	4 - 8	Yes	68.7	11.3	13.0	24.5	0.24	1.1	85.6	-1.25	-1.75	2.35
P of ANOVA sources:	EC_e			<0.001***	<0.001***	<0.001***	<0.001***	0.298 ns	<0.001***	0.182 ns	0.288 ns	0.013*	0.961 ns
	EC_w			<0.001***	<0.001***	0.001**	<0.001***	<0.001***	<0.001***	<0.001***	<0.001***	<0.001***	0.084 ns
	SL			<0.001***	<0.001***	0.762 ns	0.001**	<0.001***	<0.001***	0.003***	<0.001***	0.090 ns	0.017 *
	$EC_e \times EC_w$			0.073 ns	0.269 ns	0.935 ns	0.260 ns	0.491 ns	0.070 ns	0.041*	0.040*	0.045*	0.059 ns
	EC_e × LR			0.001**	0.173 ns	0.086 ns	<0.001***	0.011*	<0.001***	0.091 ns	0.357 ns	0.940 ns	0.571 ns
	EC_w × LR			0.831 ns	0.049*	0.131 ns	<0.001***	0.817 ns	0.009**	0.336 ns	0.460 ns	0.779 ns	0.577 ns
	$EC_e \times EC_w$ × LR			0.340 ns	0.108 ns	0.579 ns	0.002**	0.421 ns	0.097 ns	0.142 ns	0.848 ns	0.759 ns	0.549 ns

Table S2: Eigen analysis of the correlation matrix.

Number	Eigenvalue	Variation (%)	Cumulated percentage
1	10.6750	56.184	56.184
2	3.0732	16.175	72.359
3	1.8481	9.727	82.086
4	1.4130	7.437	89.523
5	0.7674	4.039	93.561
6	0.4476	2.356	95.917
7	0.2908	1.530	97.448
8	0.2556	1.345	98.793
9	0.1129	0.594	99.387
10	0.0984	0.518	99.905
11	0.0181	0.095	100.000

Table S3: Factorial loadings for principal component

	**PC1**	**PC2**	**PC 3**	**PC4**	**PC5**	**PC6**	**PC7**	**PC8**	**PC9**	**PC10**	**PC11**
DW	0.967662	0.148816	-0.010983	0.094192	0.140052	-0.096997	-0.039181	0.005485	0.010997	-0.034262	0.024659
K(R)	0.930546	-0.200449	0.106119	-0.039838	-0.054767	0.009624	0.254385	-0.030085	0.109975	0.004858	-0.015166
Mg(L)	0.910777	0.273341	0.150167	0.074925	0.036265	0.139440	-0.095477	-0.052077	0.182119	-0.025734	0.034501
RWC	0.897937	-0.119736	-0.116329	-0.294330	0.059186	0.005839	0.014164	0.249749	-0.016022	0.105695	-0.040858
WUE	0.860229	0.290757	-0.172627	0.299874	-0.204412	-0.056864	0.025755	0.014368	0.063637	0.004676	-0.076045
Ca(S)	0.847786	0.257532	-0.031091	0.299528	-0.218662	0.069180	-0.168085	0.110506	-0.133388	0.112744	0.026198
OP	0.826230	-0.424304	0.129952	-0.337600	0.039250	0.008371	-0.034265	-0.034517	-0.015576	0.035760	0.030838
WP	0.825302	-0.498506	-0.168547	-0.111965	0.115850	0.068873	0.071387	-0.035159	-0.041044	0.031374	0.047521
Ca (L)	0.791728	0.275494	0.377436	0.212764	-0.183606	-0.107290	-0.050163	-0.204918	-0.103094	-0.095763	-0.003846
R:S	0.781407	0.022748	0.173393	0.011838	0.586708	0.038854	0.027154	0.051979	-0.039485	-0.087292	-0.018088
Mg(S)	0.747473	0.528135	-0.090823	0.126582	-0.119438	0.334114	0.000845	0.094650	0.044602	-0.025828	0.023917
Mg(R)	-0.022875	0.833747	0.394703	-0.258035	0.150491	0.175205	0.077783	-0.117049	-0.034201	0.084877	-0.022413
Ca(R)	-0.298430	0.774479	0.247316	0.211398	0.254928	-0.335861	0.045215	0.148590	0.015511	0.046466	0.030866
K(S)	-0.372459	-0.462383	0.685181	0.271916	-0.002546	0.220382	-0.027626	0.203596	-0.051182	-0.101538	-0.018611
Na(L)	-0.777672	0.258455	0.331423	-0.251423	-0.304928	-0.004101	0.188368	0.150523	0.039408	-0.033811	0.039220
TP	-0.382037	0.050301	-0.642944	0.596156	0.131580	0.114343	0.222168	0.014629	-0.042587	-0.025024	0.018401
K	0.022849	-0.631918	0.489764	0.569456	0.026016	-0.094767	-0.004377	-0.030189	0.095048	0.127846	0.006370
Na	-0.899503	0.081628	-0.248215	-0.017513	0.148950	0.066148	-0.285262	0.038782	0.111998	-0.017168	-0.005975
Na	-0.910821	0.054872	0.180701	0.119641	0.149619	0.250288	0.040736	-0.136078	-0.019205	0.121620	-0.007799
